# Fluorescence-Based Classification of Caribbean Coral Reef Organisms and Substrates

**DOI:** 10.1371/journal.pone.0084570

**Published:** 2014-01-15

**Authors:** David G. Zawada, Charles H. Mazel

**Affiliations:** 1 United States Geological Survey, St. Petersburg Coastal and Marine Science Center, St. Petersburg, Florida, United States of America; 2 Physical Sciences Incorporated, Andover, Massachusetts, United States of America; University of Texas, United States of America

## Abstract

A diverse group of coral reef organisms, representing several phyla, possess fluorescent pigments. We investigated the potential of using the characteristic fluorescence emission spectra of these pigments to enable unsupervised, optical classification of coral reef habitats. We compiled a library of characteristic fluorescence spectra through in situ and laboratory measurements from a variety of specimens throughout the Caribbean. Because fluorescent pigments are not species-specific, the spectral library is organized in terms of 15 functional groups. We investigated the spectral separability of the functional groups in terms of the number of wavebands required to distinguish between them, using the similarity measures Spectral Angle Mapper (SAM), Spectral Information Divergence (SID), SID-SAM mixed measure, and Mahalanobis distance. This set of measures represents geometric, stochastic, joint geometric-stochastic, and statistical approaches to classifying spectra. Our hyperspectral fluorescence data were used to generate sets of 4-, 6-, and 8-waveband spectra, including random variations in relative signal amplitude, spectral peak shifts, and water-column attenuation. Each set consisted of 2 different band definitions: ‘optimally-picked’ and ‘evenly-spaced.’ The optimally-picked wavebands were chosen to coincide with as many peaks as possible in the functional group spectra. Reference libraries were formed from half of the spectra in each set and used for training purposes. Average classification accuracies ranged from 76.3% for SAM with 4 evenly-spaced wavebands to 93.8% for Mahalanobis distance with 8 evenly-spaced wavebands. The Mahalanobis distance consistently outperformed the other measures. In a second test, empirically-measured spectra were classified using the same reference libraries and the Mahalanobis distance for just the 8 evenly-spaced waveband case. Average classification accuracies were 84% and 87%, corresponding to the extremes in modeled water-column attenuation. The classification results from both tests indicate that a high degree of separability among the 15 fluorescent-spectra functional groups is possible using only a modest number of spectral bands.

## Introduction

Fluorescence is a trait expressed by numerous coral reef denizens, including algae, cnidarians, sponges, polychaetes, fish, crustaceans, and mollusks [1]–[3]. The source of the fluorescence is organism-dependent. Organisms that conduct photosynthesis, either directly or via endosymbiotic algae, contain chlorophyll, which fluoresces red [4]. Anemones, reef-building corals, and other cnidarians often contain additional pigments in their epithelial cells that fluoresce at a variety of wavelengths, corresponding to colors from cyan to red [2], [4]–[7]. These fluorescent pigments are homologs of the green fluorescent protein (GFP) first isolated in the north Pacific jellyfish *Aequorea victoria* [8], [9]. Fluorescence in other organisms has not received much attention and, consequently, its source is unknown in most cases. Possibilities include algae either colonizing the surface or in symbiosis, pigments, or other materials, such as chitin.

Mazel [4] was the first to systematically measure emission spectra from GFP-like pigments in the host tissues of a variety of freshly sampled cnidarian specimens across 10 different families. The results showed both cross-species similarities and intra-species variability. In some cases, corals from different families possessed similar fluorescence-emission spectra. In other instances, corals from the same genus or species fluoresced different colors or had similar emission spectral shapes, but with shifted peaks. Subsequent in situ measurements of fluorescence emission spectra confirmed intraspecific variations in color, intensity, and spectral shape [10], [11]. Additional spectral measurements, both in situ and in vivo, from several hundred Caribbean coral specimens revealed that four pigments, as defined by their spectral properties, accounted for the majority of observed fluorescent colors [12]. These pigments can be present individually or in various combinations that are not necessarily unique to a given species. Based on the approximate wavelength of their peak emission (in nm), these pigments were designated as ‘486’, ‘515’, ‘575’, and ‘685’. The first two fluorescent emissions are associated with host proteins in the GFP family. The emission with its peak at 575 nm may be associated with a GFP-like protein [13] or with phycoerythrin in symbiotic cyanobacteria [14]. The 685-nm peak is associated with chlorophyll in the symbiotic algae. The actual peak positions of the host-associated fluorescence spectra may vary by up to 10 nm in some cases. These emission spectra could also be broadly termed ‘cyan’, ‘green’, ‘orange’, and ‘red’. It should be noted that there are corals with fluorescence spectra that do not fall into any of these categories, but such instances are less common.

For reef-building corals, the physiological function of GFP-like pigments remains an open question. Hypothesized roles include providing photoprotection from ultraviolet radiation [5], enhancing photosynthesis in low-light environments [15], increasing visibility or signaling to other organisms [16] or to larval recruits [17], serving as camouflage for symbionts [18], providing supplementary antioxidant protection [19], or serving as part of an immune-and-damage-repair response [20].

Although much research involving coralline GFP-like pigments has been focused on their diversity, biochemical structure, and possible physiological role, comparatively little effort has been directed toward the potential of the fluorescence properties as an investigative tool for coral reef researchers. Myers et al. [11] showed that fluorescence spectra could be used to distinguish between pigmented and bleached corals, as well as between coral and macroalgae. Using a multispectral imaging system, Zawada and Jaffe [21] demonstrated a strong relationship between GFP-like fluorescence intensity and the physiological state of corals. They developed the GO ratio, a normalized-difference index based on the fluorescence intensity of the 515 (**g**reen) and 575 (**o**range) host pigments, that was highly correlated with the degree of bleaching in specimens of *Montastraea faveolata* induced by thermal stress. Although chlorophyll fluorescence behaved similarly, the GO ratio was resistant to contamination from other sources of chlorophyll fluorescence, such as the filamentous algae that colonized dead areas on the bleached corals. D'Angelo [22] found that for five species of corals both the composition and concentration of GFP-like pigments was regulated by the spectral composition and intensity of the incident light. This finding suggests that fluorescence might serve as an indicator of environmental conditions. Other researchers have found host-pigment fluorescence helpful in locating probable scleractinian-coral recruits, as they appear as bright dots against a dark background [23], [24]. This technique is also useful to follow the growth of the recruits until they grow large enough to identify to species level. However, Piniak et al. [23] note that coral recruits are not the sole source of small fluorescent features in the natural reef environment, so the addition of magnification and inspection under white light may be necessary to confirm their identity.

Given the apparent diversity in fluorescent pigment combinations among coral reef benthos, Hedley and Mumby [25] speculated that fluorescence might be suitable for detailed mapping. They noted that such a capability would be dependent upon a more complete understanding of the fluorescent characteristics and diversity across species. Mazel et al. [3] evaluated the potential of using fluorescence to classify benthic cover types in imagery collected with a laser line-scanning, multispectral fluorescence imaging system. This system recorded fluorescence in 3 narrow wavebands centered at 520, 580, and 685 nm, which were mapped to green, blue, and red channels of an RGB display system, respectively, for subsequent analysis. A classification scheme based on the relative responses in these channels was devised to assign each pixel in the image to one of seven categories – five functional groups, non-fluorescent targets, or unknown. The authors achieved results comparable to diver surveys identifying the same functional groups along 50-m transects.

The Mazel et al. [3] work utilized fluorescence in only three spectral bands, which was the limit of their imaging system. In this study, we explore the potential of using fluorescence as the basis for unsupervised classification of benthic organisms and substrates found in Caribbean reefs. In this context, only a small set of fluorescence spectra exists, one that encompasses multiple genera. Using fluorescence data collected from a wide variety of coral reef organisms and substrates, we investigate both the efficacy of different multispectral band sets on classification accuracy for a number of pertinent, ecological functional groups and the performance of several spectral classification methods. This effort only represents a conceptual design for exploiting the constrained set of available fluorescence spectra to complement conventional, reflectance-based, benthic classification methods.

## Methods

### Fluorescence-spectra functional groups

**Figure 1:**
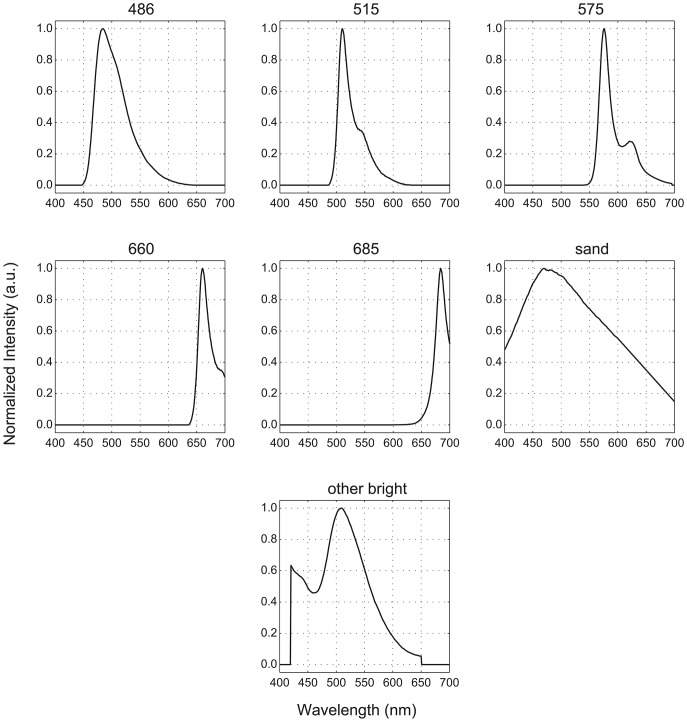
Fluorescence endmember library. Based on our measurements, most fluorescent signals observed on Caribbean reefs are attributable to one or more of these spectra.

**Figure 2:**
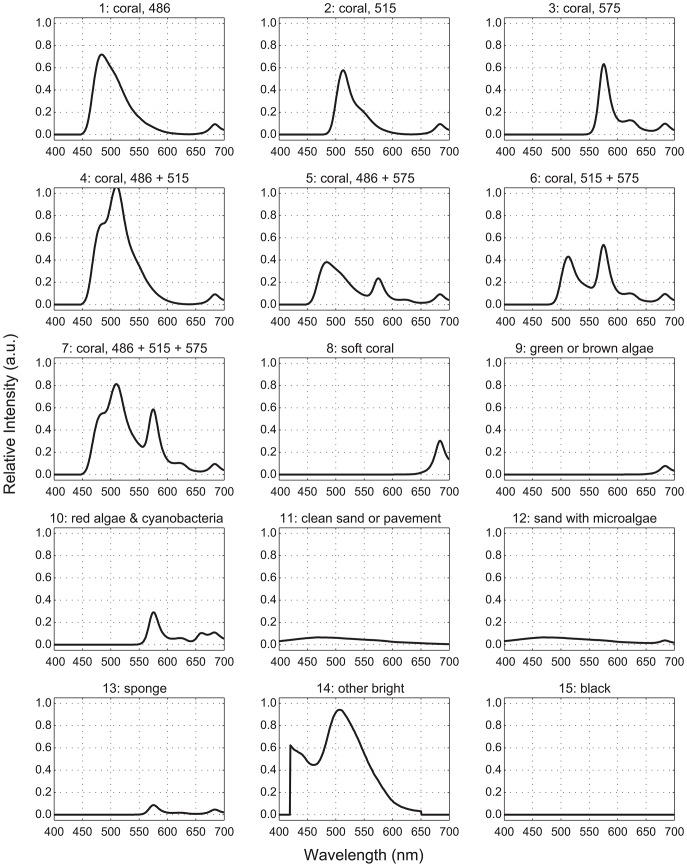
Fluorescence functional groups. These 15 spectra denote the most common fluorescent emissions we have measured on Caribbean reefs. Note: All of the stony coral functional groups (1–7) also include a chlorophyll component. Fluorescence intensities are plotted in arbitrary units, and the spectra indicate typical peak-to-peak ratios. The spectra also illustrate actual inter-functional-group differences. For example, corals with pigments 486 and 515 (group 4) tend to fluorescence ‘brighter’ than the other groups, and chlorophyll fluorescence in soft corals (group 8) is approximately 4 times more intense than that in green or brown algae (group 9). The spectra used in the classification comparisons included the effects of water-column attenuation and observed peak shifts, as described in the text.

Previous work identified 3 GFP-like pigments and chlorophyll, designated by their respective approximate peak emission wavelengths (486, 515, 575, and 685, respectively) [12] as the sources of fluorescence in most Caribbean cnidarians. The fluorescence spectra for these four pigments, plus three others – coralline sand, phycoerythrin (found in cyanobacteria and red algae), and other greenish-yellow, brightly fluorescent, non-photosynthetic organisms – formed our library of pure endmembers (Figure 1). Because many of these pigments are common to multiple organisms, it is rarely possible to uniquely associate fluorescence spectra with a particular organism. Instead, for classification purposes, we defined 15 spectral functional groups composed of endmember mixtures representing the most common fluorescence emission spectra we have measured on Caribbean reefs (Table 1 and Figure 2). One exception is the ‘486+575’ group, which we have not observed but include for completeness. The ‘soft coral’ and ‘green or brown algae’ groups do not possess any GFP-like pigments and only differ to the extent they exhibit chlorophyll fluorescence. There has been little documentation of the fluorescence of sponges, and we use a single spectrum here measured from a specimen that had associated algae exhibiting a combination of phycoerythrin and chlorophyll fluorescence.

**Table 1:** Functional groups and the pigment-mixing formulations used in the fluorescence model.

Functional Group	486	515	575	660	685	sand	other bright
1. coral, 486 only	0.4–4.0	–	–	–	0.2–1.0	–	–
2. coral, 515 only	–	0.4–4.0	–	–	0.2–1.0	–	–
3. coral, 575 only	–	–	0.4–4.0	–	0.2–1.0	–	–
4. coral, 486+515	0.4–4.0	0.4–4.0	–	–	0.2–1.0	–	–
5. coral, 486+575	0.4–2.0	–	0.4–1.0	–	0.2–1.0	–	–
6. coral, 515+575	–	0.4–3.0	0.4–3.0	–	0.2–1.0	–	–
7. coral, 486+515+575	0.4–3.0	0.4–3.0	0.4–3.0	–	0.2–1.0	–	–
8. soft coral	–	–	–	–	2.0	–	–
9. green or brown algae	–	–	–	–	0.5	–	–
10. red algae & cyanobacteria	–	–	0.5–1.5	0.5	0.5	–	–
11. clean sand or pavement	–	–	–	–	–	0.2	–
12. sand with microalgae	–	–	–	–	0.2	0.2	–
13. sponge	–	–	0.3	–	0.3	–	–
14. other bright	–	–	–	–	–	–	1.0
15. black	–	–	–	–	–	–	–

Numbers indicate relative fluorescence-peak intensities. A value within the specified range was randomly chosen for each of the 10,000 synthesized spectra for each functional group.

Random permutations of our basic mixing formulations were generated to account for the natural variability we have observed in relative fluorescence peak amplitudes (Table 1). The rules are approximate but based on our observations. For example, for a coral containing both 486 and 515 pigments plus chlorophyll, the amplitudes of the coral host-pigment peaks were permitted to vary between 0.4 and 4, while the chlorophyll was allowed to vary between 0.2 and 1. A random number generator was used to produce 10,000 combinations for subsequent testing, spanning the wavelengths of 400 nm to 700 nm. Relative amplitude combinations outside these ranges certainly exist, but the approach described here allowed for a high degree of variability, while keeping the problem tractable. The 515 nm peak in particular has been observed to vary in exact peak wavelength position more so than the other fluorescence endmembers, and the randomized peak generator also allowed this peak to vary between 500 and 520 nm.

The amplitude and spectral shape of a fluorescence emission signal arriving at a sensor will also be a function of the range to the sensor and the optical properties of the water column between the sample and the sensor. The influence of attenuation due to absorption and scattering as a function of wavelength, 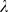, can be approximated by the vertical diffuse attenuation coefficient, 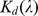. If the original spectral shape is expressed as 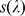, then the spectrum at some distance *d* can be computed as
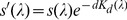
(1)where 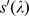 denotes the attenuated spectrum.

**Figure 3:**
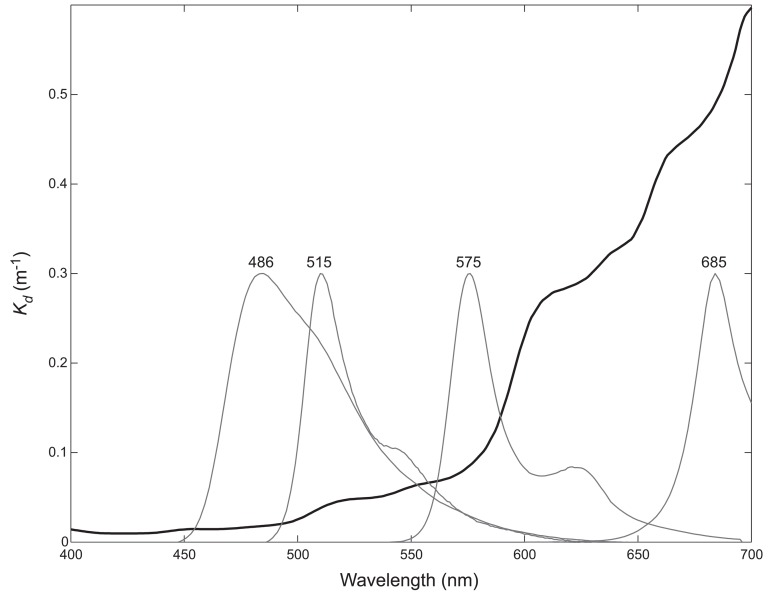
Water-column attenuation of fluorescence. The wavelength dependence of the diffuse attenuation coefficient (black) differentially attenuates the emission spectra of the pigments designated 486, 515, 575, and 685 (gray).

Figure 3 shows the emission spectra of the pigments designated 486, 515, 575, and 685 superimposed on a plot of diffuse attenuation coefficients measured at Lee Stocking Island, Bahamas, during the Office of Naval Research Coastal Benthic Optical Properties (CoBOP) research program. When a fluorescence emission corresponds to a relatively flat part of the *K_d_* curve (<∼575 nm) the spectral shape will not be altered much by this type of attenuation. Conversely, if an emission corresponds to a part of the *K_d_* curve with a steep slope (>∼575 nm) the fluorescence spectrum will be differentially attenuated, thus, altering the spectral shape. The amplitude is further affected by spherical spreading with distance, computed as
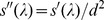
(2)where 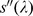 represents an attenuated spectrum used by the classification measures discussed below.

A total of 10,000 spectra per functional group were generated by randomly varying the relative amplitudes of the fluorescence components, as well as the degree of attenuation attributable to the water column and spreading due to distance. For these attenuating factors, the distance was randomly set in the range of 1≤*d*≤3 m. Half of these spectra were used as a reference library, ***R***(*g*), and the other half used as a test library, ***T***(*g*), for evaluating the chosen classification methods, where *g* denotes a specific functional group, 1≤*g*≤15.

### Spectral classification methods

Sets of 4-, 6-, and 8-bands were defined to investigate the effects of reduced spectral data on classification success. Each set consisted of 2 different band definitions: ‘optimally-picked’ and ‘evenly-spaced’ (Table 2). The optimally-picked bands were chosen to coincide with as many peaks in the functional group spectra as possible. New reference and test spectral libraries were created by applying these band definitions to the original 301-element spectra. Band values were assigned the average fluorescence intensity among the wavelengths comprising the individual bands.

**Table 2:** Different spectral band sets used in the classification tests.

Bands	Optimal 4	Even 4	Optimal 6	Even 6	Optimal 8	Even 8
Band 1	485±15	438±38	425±10	425±25	425±10	419±19
Band 2	515±10	512±37	485±15	475±25	485±10	457±19
Band 3	575±10	588±38	515±10	525±25	515±5	495±19
Band 4	685±10	662±37	575±10	575±25	550±10	533±19
Band 5	–	–	625±10	625±25	575±10	571±19
Band 6	–	–	685±10	675±25	625±10	609±19
Band 7	–	–	–	–	660±10	647±19
Band 8	–	–	–	–	685±5	685±19

Each band is designated by a center wavelength and half bandwidth, both in nm.

The impact of using reduced-band spectra was evaluated using four similarity measures: Spectral Angle Mapper, Spectral Information Divergence, SID-SAM Mixed Measure, and Mahalanobis distance. These methods represent four different approaches to spectral classification: geometric, stochastic, joint geometric-stochastic, and statistical. For consistency, the following notation is used in discussing each similarity measure. Each ***R***(*g*) and ***T***(*g*) spectral library, 1≤*g*≤15, contains a corresponding set of ***r_i_*** and ***t_i_*** spectra, 1≤*i*≤5,000, defined by Equation 2. The spectra 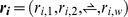 and 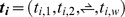 consist of *w* wavebands, such that *r_i,j_* and *t_i,j_*, 1≤*j*≤*w*, represent the fluorescence intensities at each waveband. For brevity, the subscripts have been dropped from the similarity measure definitions.

#### Spectral Angle Mapper

The Spectral Angle Mapper treats spectra as *w*-dimensional vectors. The similarity between two spectra is expressed in terms of the angle between them, computed according to
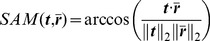
(3)where the resultant angle is in radians [26]. In Equation 3, ***t*** denotes a test spectrum from any of the test libraries and 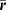 represents the average spectrum for one of the ***R***(*g*) reference libraries. *SAM* was computed between every test spectrum from all of the ***T***(*g*) libraries and the average spectrum for each ***R***(*g*) library.

#### Spectral Information Divergence

The Spectral Information Divergence is a probabilistic measure that uses relative entropy to quantify the similarity of two spectra [27]. The key concept is to regard the spectra as random variables and compute the associated probability measures describing their variability as
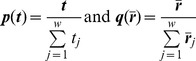
(4)where ***t*** and 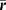 must be nonnegative, which is not an issue for fluorescence spectra. In essence, Equation 4 models the spectral information in ***t*** and 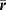 as probability distributions. Using these probability vectors, the cross-entropy between ***t*** and 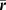 and vice versa is computed as follows
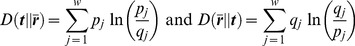
(5)where *p_j_* and *q_j_* are the *j*th components of the probability vectors ***p***(***t***) and ***q***(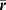) respectively. The Spectral Information Divergence is then defined as
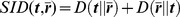
(6)which is a symmetrical assessment of the discrepancy between ***t*** and 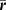 in terms of their respective probability distributions. As a result, *SID* should be more sensitive to subtle spectral variability than *SAM*, which is a purely geometrical comparator [27]. *SID* was computed for the same ***t*** and 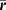 pairings used for *SAM*.

#### SID-SAM Mixed Measure

*SID*-*SAM* is a spectral similarity measure that combines the geometric characterization of *SAM* with the probabilistic discrimination of *SID* [28]. The intent of this mixed measure is to yield a more discerning result by leveraging the strengths of its two components. The *SID*-*SAM* mixed measure is computed as
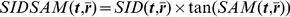
(7)where the tangent function is used as a means of calculating the perpendicular distance between ***t*** and 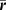, instead of projecting ***t*** onto 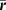 [28]. The net effect of this multiplication is to accentuate (dis)similarity extremes with respect to its components. *SIDSAM* was computed for the same ***t*** and 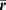 pairings used for *SAM*.

#### Mahalanobis Distance

The Mahalanobis distance is the squared-distance between a *w*-dimensional observation and the centroid of a group defined by its related *w*-dimensional members that is sensitive to the statistical distribution of those members. In the present context, the unknown observation is a given test spectrum, ***t***, with *w* bands, and the group is one of the ***R***(*g*) functional group reference libraries composed of *n* = 5,000 spectra. The Mahalanobis group represents a multidimensional, ellipsoidal space whose boundary is defined by both intra- and inter-wavelength variations exhibited by the member spectra [29]. Mathematically, the Mahalanobis distance is defined as
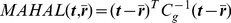
(8)where 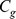 is the covariance matrix of ***R***(*g*) [30]. To ensure nonsingularity and invertability of *C_g_*, the condition *n*>*w* must be satisfied. The Mahalanobis distance was computed between every test spectrum, ***t***, from all of the test libraries and each of the ***R***(*g*) reference libraries. By using the covariance matrix, this definition of the Mahalanobis distance has the desirable property of incorporating the inherent spread in *w*-dimensional space among the *n* reference spectra in each ***R***(*g*) functional group library [30]. Thus, the resultant distances fully assimilate the extant variability in each ***R***(*g*) library of reference spectra.

For all of the similarity measures, minimum values determined the functional group classification for test spectra. *SAM*, *SID*, and *SIDSAM* were implemented in MATLAB R2011b (Mathworks), and Mahalanobis distances were computed using the *classify()* function in the MATLAB Statistics Toolbox.

### Empirical evaluations

**Figure 4:**
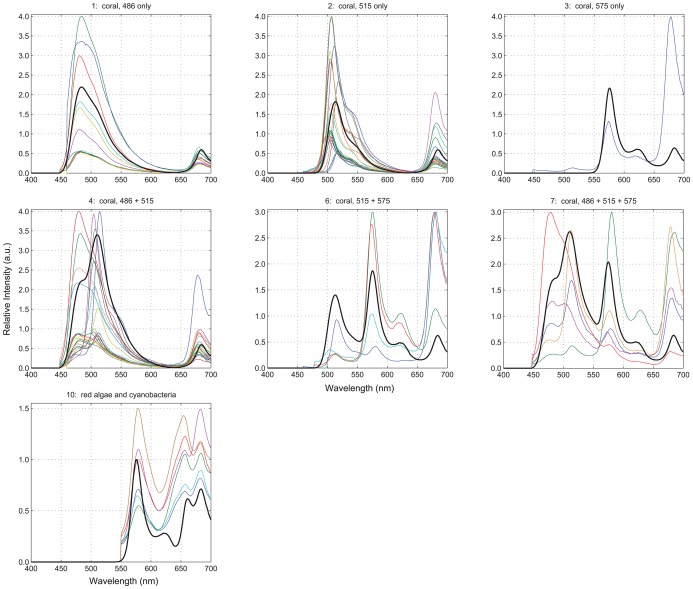
Empirically-measured spectra. This collection of actual fluorescence spectra was used to test the robustness of the Mahalanobis distance similarity measure trained with the modeled spectra. Water-column attenuation effects were applied prior to classification. Colors were used to solely improve legibility. There is no significance to multiple spectra having the same color. The thick black spectra represent the corresponding average of the 5,000 modeled spectra used as the reference set for each functional group.

Although our algorithm for synthesizing test spectra incorporates variability both in the intensity of fluorescence and the location of peak emissions, it is exceedingly difficult to account for the full extent of natural variability. To more fully test the effectiveness of fluorescence-based classification, examples of empirically-measured spectra were classified using the same reference libraries, ***R***(*g*). These spectra (Figure 4) represent the coral and red algae functional groups, which are the most challenging ones to distinguish within our dataset and the most ecologically-relevant ones for coral reefs.

Examples were chosen to represent the range in emission-peak intensities and positions that we have observed in the field. Note that in the Caribbean, ‘515’ is the predominant fluorescent GFP-like pigment and ‘575’ is the rarest. Consequently, most of the example spectra used in this test contain the ‘515’ pigment. Because the spectral measurements were made within 1 cm of the specimen, attenuation effects were applied for distances of 1-m and 3-m, yielding two spectral sets for testing. Each set was classified using the 8-band, ‘evenly-spaced’ spectral-band set with the Mahalanobis distance similarity measure.

## Results

### Modeled spectra

**Figure 5:**
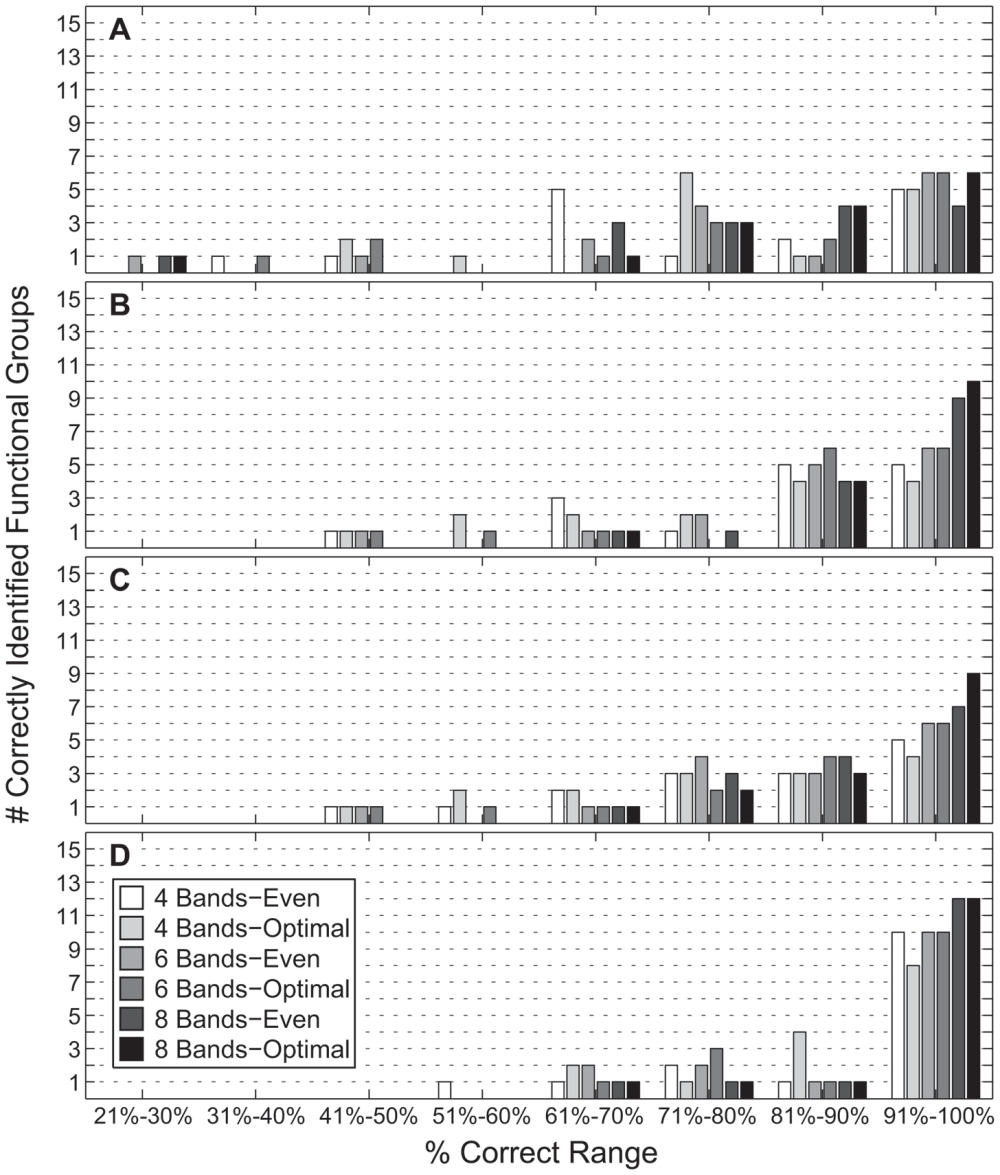
Classification accuracy histograms. Instead of plotting on a functional group basis, the results have been binned according to the percent of successful classifications. This plot indicates the overall behavior of each similarity measure to the different waveband combinations, and clearly shows the superior performance of the Mahalanobis distance (D) relative to the other measures (A-*SAM*, B-*SID*, C-*SIDSAM*).

The spectral classification results indicate that a high degree of separability among the 15 fluorescent-spectra functional groups is possible using only a modest number of spectral bands (Tables 3–6 and Figure 5). However, a few functional groups were difficult to identify, regardless of the number of bands or classification method. Distinguishing between groups 8 (‘soft coral’) and 9 (‘green or brown algae’) proved problematic, as did groups 10 (‘red algae & cyanobacteria’) and 13 (‘sponge’), particularly for the 4-band scenarios.

**Table 3:** Classification results for *SAM*.

Group	4 Bands-Even	4 Bands-Optimal	6 Bands-Even	6 Bands-Optimal	8 Bands-Even	8 Bands-Optimal
1. coral, 486 only	99.98%	98.44%	100.00%	100.00%	99.98%	99.98%
2. coral, 515 only	98.76%	90.38%	97.22%	90.96%	72.82%	97.30%
3. coral, 575 only	88.40%	83.60%	88.22%	83.58%	88.12%	88.10%
4. coral, 486+515	82.54%	73.82%	75.44%	73.94%	66.60%	74.22%
5. coral, 486+575	66.98%	71.40%	91.46%	92.02%	86.94%	92.14%
6. coral, 515+575	65.26%	71.64%	74.78%	71.72%	68.50%	74.00%
7. coral, 486+515+575	61.56%	50.26%	65.44%	64.68%	62.00%	65.00%
8. soft coral	**36.08%**	72.62%	**29.26%**	**30.28%**	72.56%	72.44%
9. green or brown algae	73.46%	73.44%	72.30%	72.30%	**27.76%**	**27.66%**
10. red algae & cyanobacteria	44.66%	47.60%	43.20%	47.98%	80.90%	84.30%
11. clean sand or pavement	100.00%	100.00%	100.00%	100.00%	100.00%	100.00%
12. sand with microalgae	64.82%	74.24%	72.86%	84.92%	78.58%	85.72%
13. sponge	62.64%	**45.36%**	65.94%	45.60%	88.90%	88.72%
14. other bright	100.00%	100.00%	100.00%	100.00%	100.00%	100.00%
15. black	100.00%	100.00%	100.00%	100.00%	100.00%	100.00%
Mean	76.34%	76.85%	78.41%	77.20%	79.58%	83.31%
SD	21.22%	18.82%	21.64%	22.14%	19.39%	19.21%

Highlighted values indicate the minimum score for each band configuration.

**Table 4:** Classification results for *SID*.

Group	4 Bands-Even	4 Bands-Optimal	6 Bands-Even	6 Bands-Optimal	8 Bands-Even	8 Bands-Optimal
1. coral, 486 only	98.12%	98.42%	100.00%	100.00%	100.00%	100.00%
2. coral, 515 only	100.00%	87.02%	91.30%	87.92%	100.00%	93.12%
3. coral, 575 only	87.08%	81.60%	86.30%	81.42%	86.00%	85.90%
4. coral, 486+515	88.12%	81.82%	82.62%	81.96%	85.80%	83.74%
5. coral, 486+575	79.54%	53.10%	96.08%	97.46%	93.00%	98.00%
6. coral, 515+575	89.32%	86.74%	88.24%	88.24%	91.78%	90.66%
7. coral, 486+515+575	82.10%	62.98%	79.54%	80.70%	84.10%	83.32%
8. soft coral	60.20%	67.36%	61.60%	61.36%	**63.58%**	**62.54%**
9. green or brown algae	80.22%	77.04%	80.86%	80.50%	79.52%	80.98%
10. red algae & cyanobacteria	**44.68%**	52.36%	**43.32%**	53.48%	88.00%	95.48%
11. clean sand or pavement	100.00%	100.00%	100.00%	100.00%	100.00%	100.00%
12. sand with microalgae	68.88%	71.22%	82.98%	100.00%	92.52%	100.00%
13. sponge	68.66%	**45.90%**	78.04%	**47.60%**	100.00%	100.00%
14. other bright	100.00%	100.00%	100.00%	100.00%	100.00%	100.00%
15. black	100.00%	100.00%	100.00%	100.00%	100.00%	100.00%
Mean	83.13%	77.70%	84.73%	84.04%	90.95%	91.58%
SD	16.65%	18.41%	15.73%	17.52%	10.30%	10.70%

Highlighted values indicate the minimum score for each band configuration.

**Table 5:** Classification results for *SIDSAM*.

Group	4 Bands-Even	4 Bands-Optimal	6 Bands-Even	6 Bands-Optimal	8 Bands-Even	8 Bands-Optimal
1. coral, 486 only	99.70%	98.42%	100.00%	100.00%	99.98%	100.00%
2. coral, 515 only	100.00%	88.40%	93.26%	89.26%	100.00%	95.18%
3. coral, 575 only	87.36%	82.32%	86.88%	82.28%	86.66%	86.60%
4. coral, 486+515	87.62%	78.48%	79.96%	78.66%	80.00%	79.92%
5. coral, 486+575	79.00%	56.56%	94.90%	96.68%	92.86%	96.46%
6. coral, 515+575	86.14%	85.30%	84.82%	85.96%	88.22%	87.46%
7. coral, 486+515+575	76.30%	61.00%	75.02%	76.02%	78.38%	77.62%
8. soft coral	58.44%	69.10%	61.32%	60.92%	**63.94%**	**63.06%**
9. green or brown algae	77.56%	75.84%	80.68%	80.18%	79.62%	81.06%
10. red algae & cyanobacteria	**44.66%**	50.82%	**43.24%**	51.80%	85.68%	91.70%
11. clean sand or pavement	100.00%	100.00%	100.00%	100.00%	100.00%	100.00%
12. sand with microalgae	67.58%	72.12%	79.70%	96.80%	87.80%	97.08%
13. sponge	66.68%	**45.64**%	75.60%	**46.74%**	98.68%	98.08%
14. other bright	100.00%	100.00%	100.00%	100.00%	100.00%	100.00%
15. black	100.00%	100.00%	100.00%	100.00%	100.00%	100.00%
Mean	82.07%	77.60%	83.69%	83.02%	89.45%	90.28%
SD	17.20%	18.32%	16.06%	17.80%	10.82%	10.90%

Highlighted values indicate the minimum score for each band configuration.

**Table 6:** Classification results for *MAHAL*.

Group	4 Bands-Even	4 Bands-Optimal	6 Bands-Even	6 Bands-Optimal	8 Bands-Even	8 Bands-Optimal
1. coral, 486 only	100.00%	99.86%	100.00%	99.86%	100.00%	99.98%
2. coral, 515 only	99.50%	90.90%	94.16%	91.36%	100.00%	93.74%
3. coral, 575 only	86.96%	82.32%	84.40%	79.44%	92.32%	95.30%
4. coral, 486+515	94.60%	97.18%	99.46%	97.40%	99.98%	99.84%
5. coral, 486+575	91.32%	89.86%	91.72%	90.88%	92.68%	91.00%
6. coral, 515+575	97.54%	90.62%	92.58%	90.90%	100.00%	92.78%
7. coral, 486+515+575	95.88%	97.90%	98.52%	98.04%	99.98%	99.92%
8. soft coral	65.70%	**66.10%**	**66.00%**	**66.16%**	**66.08%**	**66.58%**
9. green or brown algae	**56.58%**	77.90%	66.36%	77.90%	75.68%	78.08%
10. red algae & cyanobacteria	72.14%	69.72%	77.88%	72.70%	93.96%	100.00%
11. clean sand or pavement	99.06%	86.24%	99.72%	100.00%	100.00%	100.00%
12. sand with microalgae	100.00%	94.62%	100.00%	100.00%	100.00%	100.00%
13. sponge	78.36%	81.24%	78.90%	82.16%	86.80%	86.84%
14. other bright	100.00%	94.96%	100.00%	100.00%	100.00%	100.00%
15. black	100.00%	100.00%	100.00%	100.00%	100.00%	100.00%
Mean	89.18%	87.96%	89.98%	89.79%	93.83%	93.60%
SD	14.26%	10.61%	12.31%	11.36%	10.33%	9.82%

Highlighted values indicate the minimum score for each band configuration.

For a given number of bands, the choice of ‘evenly-spaced’ versus ‘optimally-picked’ waveband locations had less of an impact on classification success than the type of similarity measure. *SAM* (Table 3) consistently performed the worst and *MAHAL* (Table 6) the best. This pattern is evident when the results in Tables 3–6 are plotted as a histogram (Figure 5). For *SAM*, less than half of the 15 functional groups are correctly identified at the 90+% level, whereas *MAHAL* correctly identifies at least 10 functional groups at the 90+% level for all band configurations except for the 4-band optimal case. In fact, *SAM* and *MAHAL* perform rather consistently, regardless of the spectral band configuration, whereas *SID* shows significant improvement for the two 8-band test cases (Figure 5B).

In terms of average percent success across all functional groups, *SAM* steadily improved from 76.3% to 83.3% with the increasing number of bands, except for a slight decrease for the ‘6 Bands-Optimal’ set. Likewise, *MAHAL* performed better with more bands, yielding mean correct classifications between 88.0% and 93.8%. Interestingly, performance consistently dropped for each of the ‘optimal’ band sets. The remaining methods, *SID* (Table 4) and *SIDSAM* (Table 5), performed better than *SAM*, but not as well as *MAHAL*. For these two similarity measures, there is a striking drop in the mean classification success for the ‘4 Bands-Optimal’ set, primarily attributable to the poor scores for functional groups 13 (‘sponge’), 10 (‘red algae and cyanobacteria’), and 5 (‘coral, 486+575’).

### Empirically-measured spectra

With the exceptions of groups 1 (‘coral, 486 only’) and 7 (‘coral, 486+515+575’), the empirical spectra were successfully classified to a degree comparable to that achieved for the modeled spectra (Table 7). Of the 13 misidentifications for the 1-m detection distance, 9 (69%) were incorrectly assigned to group 4 (‘coral, 486+515’). Increased water-column attenuation did not affect the outcome for 4 of the 7 sets of empirical spectra. Results for groups 1 (‘coral, 486 only’) and 2 (‘coral, 515 only’) improved at a 3-m detection distance, but were slightly worse for group 4. Average classification success was 84% for the 1-m attenuation case and 87% for the 3-m case.

**Table 7:** Classification results for empirically-measured, fluorescence spectra.

			Detection distance = 1 m		Detection distance = 3 m	
Group	*N*	# Genera	# Correct (%)	Misidentified Group(s)	# Correct (%)	Misidentified Group(s)
1. coral, 486 only	13	3	6 (46%)	4	8 (62% *)	4
2. coral, 515 only	23	7	20 (87%)	4, 6, 7	23 (100% *)	-
3. coral, 575 only	1	1	1 (100%)	-	1 (100%)	-
4. coral, 486+515	21	6	20 (95%)	1	19 (90% *)	1
6. coral, 515+575	4	2	4 (100%)	-	4 (100%)	-
7. coral, 486+515+575	5	2	3 (60%)	4, 6	3 (60%)	4, 6
10. red algae & cyanobacteria	6	4	6 (100%)	-	6 (100%)	-
Mean			84%		87%	
SD			22%		19%	

Water-column attenuation effects were applied for distances of 1 m and 3 m. Different numbers of example field spectra were used for each functional group. Example spectra from multiple genera were used for each group when possible. Erroneous classifications are indicated by the corresponding functional-group number. Asterisks denote groups for which classification success changed with increasing attenuation effects.

## Discussion

In this study we have explored the potential to use only a few broad spectral bands to distinguish between functional groups of ecological significance in the coral reef environment. This approach is made possible by the constrained set of fluorescence spectra present among Caribbean reef organisms. A reflected light signal always has contributions throughout the visible spectrum, with a dependence on three spectrally variable factors - the inherent reflectance of the subject, the downwelling light, and the water column optical properties. In contrast, the fluorescence spectrum emitted from a given pigment is independent of the excitation wavelength, reducing the potential signal variability.

The functional groups in our spectral library represent the most common fluorescence spectra we have measured to date from a wide variety of reef organisms throughout the Caribbean, including algae, corals, and coralline sand (Figure 2). Our use of functional groups, rather than genus or species, was necessitated by the small palette of fluorescent pigments shared among reef denizens and by inter-species variability [3].

To evaluate the effectiveness of multispectral, fluorescence-based classification, we used both modeled and empirically-measured spectra. In the modeled approach, spectra for each functional group were synthesized from appropriate mixtures of 7 endmember fluorescence spectra (Figure 1). This strategy enabled us to include thousands of hypothetical combinations of emission-peak amplitudes and positions and of attenuation effects, more than would be possible to obtain from direct measurements. Despite this diversity of spectra, functional groups were successfully identified by using only a few broad spectral bands. An average classification accuracy of nearly 90% was attained with just 4 bands that evenly divided the visible spectrum (Table 6). Consequently, accurate, fluorescence-based classification is achievable with a multispectral sensor equipped with broadband filters. There is no compelling need for expensive, narrow-band filters or a more complex hyperspectral alternative to separate these predominant fluorescence spectra.

Among our four similarity measures, the Mahalanobis distance consistently yielded the best results, regardless of the waveband configuration. This superior performance is attributable to the fact that both the intraband variance and interband covariance of a training set are used to compute the Mahalanobis distance [29], [31]. Hence, the full range of spectral variability in the reference libraries resulting from our random permutations of fluorescence peak position and amplitude, in combination with attenuation effects, was used to classify an unknown spectrum as a member of a particular functional group. In contrast, the other methods compared unknown spectra to an average reference spectrum representing each functional group. Also noteworthy, those methods were developed for classifying hyperspectral data, not the 4- to 8-band spectra used in this study.

All of the evaluated similarity measures struggled to distinguish between groups 8 (‘soft coral’) and 9 (‘green or brown algae’) and, to a lesser extent, between groups 10 (‘red algae & cyanobacteria’) and 13 (‘sponge’). This difficulty is attributable to spectral similarity, especially for groups 8 and 9 which only exhibit chlorophyll fluorescence (Figure 2). In creating the reference libraries, random perturbations were applied to the canonical fluorescent emission spectra for each functional group to account for common attenuating factors, including detection range, water column absorption, and spherical spreading. With magnitude of the chlorophyll fluorescence peak being the only differentiating factor for groups 8 and 9, the random perturbations produced overlap in the respective reference libraries, thus, reducing the number of successful classifications for these functional groups.

To more rigorously test our fluorescence-based classification technique, we compared empirically-measured spectra to the same reference library of modeled spectra. Similar classification accuracies were achieved for most groups, indicating that our model generally accounted for natural variability. The frequency of groups 1 (‘coral, 486 only’) and 7 (‘486+515+575’) being mistakenly identified as group 4 (‘486+515’) likely stems from fluctuations in the broadness of the 486-nm emission and spectral shifts in the emission peak of ‘515’ that were not adequately characterized by our simple band definitions. As in the modeled-spectra results, water-column attenuation, over our specified range, did not significantly hinder classification success. This finding further underscores the importance of sufficiently capturing the spectral shape of fluorescent emissions. Given the near ubiquity of chlorophyll fluorescence, an improved model might ignore this portion of the spectrum and place greater emphasis on shorter wavelengths.

The results of this study demonstrate the under-utilized value of fluorescence as a distinguishing factor for classifying Caribbean reef habitats. As in prior studies [3], [11], we demonstrated the separability of stony coral, algae, and sand based on their respective fluorescence spectra. More importantly, we showed that fluorescence enables a finer degree of classification within each of those broad categories, and that the Mahalanobis distance is a robust classification method for fluorescence spectra.

Notwithstanding these strengths, there are challenges to acquiring fluorescence spectra. No off-the-shelf, commercial instrumentation currently exists, so interested researchers must either develop their own or borrow the necessary equipment. In addition, inefficiencies of the fluorescence process coupled with water column attenuation, especially for wavelengths of ∼570 nm and longer, result in low signal strengths. Consequently, detection necessitates underwater ‘remote sensing’ approaches at distances typically on the order of a few meters. Notably, Mazel et al. [3] achieved a 7 m detection distance using a prototype Fluorescence Imaging Laser-Line Scanner (FILLS) that has been operated both in towed and submersible-mounted modes. However, the logistical and operational costs of FILLS undermine its feasibility as a general purpose tool. Restricting detection distances to <10 m correspondingly restricts the swath width to a similar limit. For example, FILLS data sets spanned a nominal 10 m swath width.

Cognizant of these factors, we designed this study to accommodate them and to address the feasibility of building an underwater, multispectral, fluoro-sensing instrument based on commercially-available technology. The detection distances used in generating our reference and test spectral libraries were randomly set between 1 and 3 m, and subsequently used to estimate water-column attenuation effects. The core components for a suitable instrument are sensitive photodectectors, optical filters, and a fluorescence-excitation light source. Both photomultiplier tubes (PMTs) and monochromatic, scientific-grade CCD cameras have successfully been used to quantify fluorescence in coral reef habitats [3], [21]. With the maturation of the machine-vision field, numerous compact, high-resolution, digital cameras of sufficient sensitivity are readily available. Grayscale cameras can be coupled with desired bandpass filters to record the fluorescence signal of interest without the confounding effects of Bayer filtering used in color cameras. Multiple fluorescence emissions can be recorded by using multiple cameras, a filter wheel, or multiple beamsplitters that map to separate portions of the CCD. Exciting the full palette of fluorescent pigments requires a blue light source that emits in the 440 nm to 470 nm range, and a system could benefit from rapid sequential imaging with more than one excitation wavelength for increased efficiency and signal strength. Although broad-spectrum strobes with appropriate filters have been used [21], more efficient and powerful blue LEDs are a better option, especially for detection ranges >1 m. In addition, a variety of small-footprint, solid-state lasers exist, which possibly could be used to build a compact, practical version of FILLS. Such an instrument theoretically could measure both multispectral fluorescence and seafloor topography, providing a textural component to improve classification algorithms. A hypothetical detection system could also include the capability to measure the range to the sample and the local water optical properties, thus, enabling real-time correction for spectral attenuation effects.

Within our specified limits for detection distances, several underwater ‘remote sensing’ modalities have been used in coral reef research, including SCUBA divers, towed vehicles, and autonomous underwater vehicles (AUVs). One appealing, diver-based system is the Low-Light-Level Underwater, Multispectral Imaging System-2 (LUMIS-2; http://jaffeweb.ucsd.edu/node/246), which is the next generation of the system used by Zawada and Jaffe [21] to investigate the effects of bleaching on coral fluorescence intensity. LUMIS-2 is a more compact, flexible instrument capable of fluorescence imaging in 3 discrete spectral bands, plus composite RGB reflectance imaging. The system incorporates high-power, blue LED strobes to stimulate fluorescence and conventional white-light strobes for reflectance imaging. A number of towed vehicles are commercially available that could be equipped with the necessary fluorescence-recording instrumentation. One example is the Deep Along-Track Reef-Imaging System (Deep ATRIS), which currently includes a gigabit-ethernet, CCD camera, white lights, and an obstacle-avoidance system (http://ngom.usgs.gov/dsp/tech/deep_atris/index.php) [32]. Measuring 0.81 m in length and weighing 45 kg in air, Deep ATRIS is typically deployed from a 7.6-m boat and has been used to survey reefs in both Biscayne National Park and Dry Tortugas National Park, FL. Dual sonars enable Deep ATRIS to continually monitor its altitude and sense obstructions. The system dynamically maintains a user-specified altitude. Various AUVs, both commercial and custom-made, have been used to either acoustically or optically image reefs [33]–[35], and could conceivably be configured for reef fluorescence work. Units such as the Sirius have been equipped with an assortment of sensors, including high-resolution stereo cameras, multibeam sonar, CTD, and a chlorophyll fluorometer, and have demonstrated the ability to operate at an altitude of 2 m above the reef [34].

In our view, fluorescence provides an alternative, yet complementary, approach to conventional, reflectance-based classification of coral reef habitats. Although reef-related fluorescence spectra cannot be acquired via airborne or spaceborne instruments, which provide synoptic coverage, there are advantages to close-range, in situ ‘remote sensing’ approaches that improve classification success. One gains higher spatial resolution, allowing fluorescence spectra potentially to be collected over millimeter or finer spatial scales, essentially at the coral polyp level, which decreases sub-pixel signal mixing. In addition, short, in situ operating distances eliminate atmospheric effects and significantly reduce water-column attenuation. Reflectance measurements acquired in a corresponding manner would similarly benefit. Dual fluorescence-reflectance approaches, comparable to LUMIS-2, are promising improvements to coral-reef mapping techniques. Moreover, designing such an instrument for deployment from a towed vehicle would allow substantial large areas to be mapped in a reasonable time period. For example, Deep ATRIS has been towed at 3 m s^−1^ over coral reef habitats. At that speed, roughly 11 km of transect could be mapped in one hour. Finally, besides improving classification results, using fluorescence also offers the potential for other diagnostic measurements, such as stress levels as demonstrated by Zawada and Jaffe [21].
